# Supplementary material for: Are carbon emissions trading and green financial instruments synergistic? -Comprehensive quantitative research based on content analysis
Source: PLoS One. 2024 Mar 7;19(3):e0298601. doi: 10.1371/journal.pone.0298601 (PMC10919601; doi:10.1371/journal.pone.0298601)
Supplement: S1 Data — (ZIP) [file pone.0298601.s001.zip › Data/地级市Z_F工作报告.zip!/╔┬╬≈/╔╠┬σ╩╨/└┤╫╘╣1⁄2╓┌║┼í╛┬φ┐╦╩2╛▌═°í┐_2017.docx]

各位代表：

现在，我代表市人民政府向大会报告工作，请予审议，并请市政协委员和列席人员提出意见。

一、2016年和过去五年工作回顾

2016年是“十三五”规划开局之年，也是商洛追赶超越的重要一年。一年来，在省委、省政府和市委坚强领导下，我们团结带领全市人民顽强拼搏、攻坚克难，较好完成了市三届人大六次会议确定的各项目标任务，实现了“十三五”开门红。全年完成生产总值699.3亿元、增长10%，增速居全省前列；城乡居民收入分别达到25468元和8358元，增长8.3%和8.5%，增速分别位居全省第1位和第2位。重点做了以下工作：

（一）促投资稳增长，经济运行稳中有进。持续增加有效投资，160个市级重点项目完成投资453.6亿元。必康医药产业园二期、比亚迪锂离子电池电解液等65个项目基本建成，在建亿元以上项目达到340个，完成全社会固定资产投资935.55亿元、增长21.9%。出台工业稳增长10条政策措施，推进企业稳产促销，完成规模以上工业增加值247.47亿元、增长15.1%，增速位居全省第2位，规模企业总数达到233户，主要工业品产销率达到94.8%。不断扩大三产消费，旅游休闲消费持续攀升，以网络购物、信息消费为代表的新兴消费蓬勃发展，实现社会消费品零售总额174.93亿元、增长13.1%。

（二）调结构促转型，产业升级亮点纷呈。工业结构持续优化，绿色食品、生物医药产值分别增长34.9%、28.1%，新能源、电子信息、智能制造等战略性新兴产业增加值增长16%，矿业占规模工业比重下降3个百分点。现代农业加快发展，新建省、市级现代农业园区16个，新认定无公害农产品产地28个；生态农业成为全省亮点，核桃、板栗、中药材、香菇、鸡蛋产量稳居全省第一，粮食实现“五连丰”。电子商务、物流快递、健康养老等现代服务业快速兴起，电商交易额增长48%，快递业务收入增长96%。

（三）打基础强支撑，协调发展成效明显。中心城区功能不断完善，“十大城建”“十大棚改”工程有序推进，90个重点建设项目完成投资245亿元，国家卫生城市、园林城市、森林城市创建全面启动，市区城市管理体制逐步理顺。县城扩容提质进程加快，完成投资16.9亿元，全市户籍人口城镇化率达到51.5%。基础设施建设不断加强，完成国省干线和农村公路建设改造558公里，解决6万人饮水安全问题，商州至洛南、商南天然气长输管线开工建设。生态环境建设成效显著，完成造林绿化25万亩，治理水土流失700平方公里，主要河流出境断面水质稳定在Ⅱ类以上，中心城区空气质量优良以上天数达到310天、高居全省第一，五项主要污染物减排任务超额完成。

（四）抓改革扩开放，发展活力有效释放。有序推进供给侧结构性改革，淘汰落后产能20万吨，商品房去库存7074套，为企业争取直供电9亿度，节省电费2000余万元。深入推进“放管服”改革，“三个清单”落实到位，累计取消下放行政审批事项166项，精简率46%。“五证合一、一照一码”全面实施，新登记私营企业数量增长23.5%，非公经济增加值占GDP比重达到54.2%。投融资体制、国企改革取得重要进展，城投、交投、园区开发公司和融资担保公司等市级五家国有企业投融资能力不断增强，81个PPP项目进入省级储备库、6个付诸实施。天之润、百盛丝绸、正太能源等7家企业在陕西股权交易中心挂牌。建成市级外贸出口示范基地4个，外贸进出口总额14.42亿元。招商引资到位资金515亿元，增长19.5%。

（五）抓脱贫惠民生，人民获得感不断增多。“两个80%”全面落实，全年民生支出157.3亿元，较上年增加6.3亿元。完成移民搬迁3.18万户，20.41万贫困人口实现稳定脱贫。新增城镇就业1.89万人，农村劳动力转移就业53.9万人，企业退休人员养老金实现“十二连增”。13年免费教育全面落实，医药卫生体制改革有序推进，分级诊疗制度和全面两孩政策有效实施。《带灯》《沉香》等精品剧目亮相全国舞台，《最美商洛》风光片获全国优秀奖。体育器材配送在全省率先实现镇办、社区全覆盖，镇安籍运动员张丽在15届里约残奥会上勇夺4金，为祖国和家乡赢得了荣誉。平安商洛建设深入推进，生产安全、环境安全、网络安全、食品药品安全监管力度不断加大，治安防控体系日益完善，社会大局和谐稳定。

2016年，我们不断加强政府自身建设，严格落实全面从严治党各项部署，扎实开展“两学一做”学习教育，公务人员“四个意识”显著增强。自觉接受市人大法律监督、工作监督和市政协民主监督，认真办理市人大代表建议176件、政协委员提案239件。重视审计监督、舆论监督和社会监督，积极回应社会和群众关切，赢得了各方信任和支持。同时，国防动员、民族宗教、外事侨务、防震减灾、妇女儿童、气象、水文、档案、统计、人防、方志、老龄、无线电及慈善、红十字会、残疾人事业、关心下一代等工作，都取得了新成绩。

各位代表，2016年各项任务的完成，为本届政府五年任期画上了圆满句号。过去五年，全市上下凝心聚力、风雨同舟，在应对挑战中保持定力、蓄势挖潜，在拼搏进取中借势发力、争先进位，付出了艰辛努力，取得了丰硕成果，为同步够格建成小康社会，奠定了坚实基础!

这五年，是商洛经济换挡提速、实现跨越发展、填补多项历史空白的五年。经济年均增长11.9%，高于全省2.1个百分点，总量五年增长1.9倍，人均生产总值达到4315美元。商洛电厂、镇安抽水蓄能电站等事关长远发展的一批重大项目相继开工建设，光伏全产业链成为全省典型，新能源汽车、智能制造等新兴产业快速成长，钒氮合金产量世界第一，金川封幸皂素清洁生产工艺国内首创，商丹园区顺利晋级为省级高新区，陕西炼石、必康制药成功借壳上市，20余家国内外500强企业落户商洛。

这五年，是商洛突破交通瓶颈、走出大山、融入西安迈进关天经济圈的五年。西康、宁西两条铁路复线和西商高速二线建成通车，包茂、沪陕、福银三条高速贯穿全境，山柞高速全线开工建设，全市高速公路通车里程达到409公里，率先在全省实现县县通高速，西武、西渝高铁列入国家“十三五”规划，即将付诸实施，昔日交通瓶颈已成为竞争新优势。

这五年，是商洛完善城镇功能、统筹协调发展、城乡面貌发生巨大变化的五年。“一体两翼”中心城市初具规模，环城北路、商鞅大道、市一级汽车客运站、市体育中心建成投用，商丹和商洛城市干线、市文化艺术中心、新建商中、滨江银杏公园等项目基本完工，规划区建成面积达到54.5平方公里。柞水、商南两县成功创建“国家卫生县城”，省级卫生城市、园林城市创建实现县区全覆盖。12个镇入列全国重点镇，漫川关、云盖寺、棣花、凤凰等特色小镇享誉省内外，建设乡村旅游示范村17个，朱家湾、前店子荣获“中国最美休闲乡村”，宜游、宜居、宜业的山水园林和生态休闲城市雏形显现。

这五年，是商洛生态环境不断优化、旅游产业悄然兴起、“秦岭最美是商洛”唱响打靓的五年。森林覆盖率达到67%，五年提高近5个百分点，跃居全省第一，洛南、商南两县成为全国绿化模范县。流域治理“河长制”经验在全省推广，保障了“一江清水供京津”。空气质量连年稳居全省第一，“商洛蓝”刷爆朋友圈。商南金丝峡成为陕南首家5A级景区，对外开放旅游景区45家，其中3A级以上26家，商洛秦岭生态旅游节、环秦岭自行车赛上升为国家级节赛，我市成功跻身“中国旅游百强市”。

这五年，是商洛民生事业发展、社会和谐稳定、人民群众幸福指数显著提升的五年。累计民生支出669亿元，城乡居民收入年均增长11.1%和12.8%。“三带一创”和“双包双促”精准扶贫模式走在全国、全省前列，58.83万贫困人口稳定脱贫，10.48万户38.51万人依靠移民搬迁走出深山，8万户居民通过安居工程喜迁新居，城镇人均住房面积33平方米、高于全省平均水平。城镇新增就业8.52万人，农村劳动力转移就业254万人次。率先在全省整市通过国家义务教育发展基本均衡认定，高考二本以上上线率连年稳居全省前列。“六五”普法圆满完成，连续五年荣获全省社会治安综合治理优秀市，法治信访经验在全省推广，公众安全感保持全省领先。

各位代表，五年砥砺奋进，成绩来之不易，得益于省委、省政府和市委的正确领导，得益于市人大、市政协的监督支持，更是全市人民凝心聚力、团结拼搏的结果。在此，我代表市政府，向全市人民、驻商部队指战员、武警官兵和公安干警，向大力支持政府工作的各位代表、各位委员和社会各界人士，表示衷心感谢并致以崇高敬意！

在总结成绩的同时，我们也清醒地看到，发展中还面临诸多困难和问题：全市经济总量偏小，部分经济指标在全省排名靠后；产业结构不尽合理，三产占比较小；地方财政困难，人均财力较低，债务负担较重；创新发展活力不足，社会公共服务水平有待提高等等。认识这些差距，我们才能自我加压、激发斗志，在全面建成小康社会的新征程中迎难而上、奋力拼搏！

二、今后五年目标任务

各位代表，展望未来，尽管前进道路上充满艰辛和挑战，但也蕴含着许多重大机遇和优势。从国家战略看：国家实施西部大开发和“一带一路”战略、加快振兴川陕革命老区、深化东西部扶贫协作、支持加工贸易向中西部转移。作为连接“一带一路”和长江经济带的重要节点城市，商洛在深度融入国家发展大战略、大格局中，大有可为、大有作为。从全省布局看：我省打造内陆改革开放新高地，加快推进陕西自贸区、丝绸之路新起点、秦岭国家公园和关中平原城市群建设，我们身在其中、利在其中。特别是省政府将西商一体化纳入全省发展战略，这对促进西商优势互补、带动商洛发展迈上快车道，必将产生更直接、更快捷的作用。从我们自身看:随着西武、西渝高铁的建设开通，商洛将进入高铁时代，全面融入西安半小时经济圈，交通区位优势将进一步转化为经济发展优势；我市林茂水丰、生态优越，空气负氧离子浓度高达2万个以上，是世界空气清新标准的20倍，山清水秀、宜居康养；我市目前已发现矿产资源60余种、大中型矿床39个，特别是新探明的洛南紫绿玛瑙为陕西增添新矿种，镇安钨钼资源储量巨大，丹凤石墨探明储量达1000万吨，山阳夏家店特大金矿远景储量超过200吨，全市矿产资源潜在价值超过5000亿元，开发利用前景广阔。

除了以上这些客观优势，我们还有勤劳质朴、穷则思变、热爱商洛、期盼发展的251万父老乡亲，这是我们追赶超越的最大优势、最大资本和最大动力源泉。有了这个优势，一切困难都能克服；有了这个资本，任何瓶颈都能突破；有了这个动力，我们的宏伟目标就一定能够实现！

各位代表，中央经济工作会对以供给侧结构性改革为主线的各项工作作出了全面部署，省委、省政府就“追赶超越”提出了具体要求，市第四次党代会描绘了“三个商洛”宏伟蓝图，全市“十三五”规划确定了未来发展的任务目标，我们将坚决贯彻中央、省委和市委部署，坚持既定思路和发展目标不动摇，坚定不移抓发展、促落实、惠民生。

今后五年政府工作的总体要求是：全面贯彻党的十八大和十八届三中、四中、五中、六中全会精神，以邓小平理论、“三个代表”重要思想、科学发展观为指导，深入贯彻习近平总书记系列重要讲话精神，坚持以脱贫攻坚统揽经济社会发展全局，以“追赶超越”、绿色发展为主线，走产业绿色化、城镇景区化、田园景观化之路，接力打好精准脱贫、现代工业、特色农业、全域旅游、新型城镇化“五大攻坚战”，倾力打造秦岭休闲之都、丝路产业新城，奋力建设创新美丽幸福商洛，全面建成小康社会。

主要预期目标是：生产总值达到1300亿元，财政总收入达到100亿元左右、地方财政收入达到60亿元，累计完成全社会固定资产投资7000亿元，力争实现“三个领先、四个超越、五个追赶”。

“三个领先”是：生态环境质量、循环经济发展、全域旅游继续保持全省领先。

“四个超越”是：生产总值、城镇居民收入、农村居民收入增速和非公经济增加值占GDP比重超过全省平均水平。

“五个追赶”是：人均GDP、常住人口城镇化率、科技贡献率、服务业和战略性新兴产业增加值占GDP比重，接近或达到全省平均水平。

未来五年，我们将全力以赴打好“五大攻坚战”：

（一）以“七个一批”为抓手，打好精准脱贫攻坚战。在积极争取中省专项资金的同时，我们市财政也要勒紧腰带、挤出资金，按照“六个精准”的要求，综合采取发展产业、转移就业、易地搬迁、生态补偿、教育扶持、健康保障、社会兜底等措施，统筹规划、聚力推进，确保2018年商南、柞水脱贫摘帽，2019年商州、洛南、丹凤、山阳、镇安脱贫摘帽，2020年全市29.68万建档立卡贫困人口全部脱贫。让商洛百姓生活的更加幸福、更有尊严！

（二）以转型升级和园区建设为抓手，打好现代工业攻坚战。坚持保护生态、循环发展理念不动摇，整装勘查、整合资源、整体利用矿产资源，推进矿产业、生物医药、绿色食品等产业转型升级、提质增效，延长产业链，提高附加值。加大招商引资力度，发挥园区聚集引领作用，依托资源、依靠科技，加快发展新材料、新能源、先进制造、电子信息等战略性新兴产业。鼓励支持企业上市，积极推进山阳中医药、商南硅科技、丹凤石墨、镇安钨钼、洛南紫绿玛瑙等高附加值产业园区建设。工业增加值年均增长13%以上，到2021年，战略性新兴产业增加值占GDP比重达到16%以上。让我们这个传统的农业市，整体迈上新型工业化的强市富民之路！

（三）以品牌战略和龙头带动为抓手，打好特色农业攻坚战。积极建设秦岭生态农业示范市，争创国家农业可持续发展试验示范市，做大做强核桃、板栗、中药材、秦岭生态茶、山菌、生态猪、土鸡、土蜂等特色产业。积极培育龙头企业、合作社、家庭农场等新型农业经营主体，全产业链带动农业产业转型升级。加强市场建设，创建知名品牌，推动农村一二三产业融合发展。到2021年，把“商洛核桃”发展成继“渭北苹果”之后我省又一标志性品牌，把商洛打造成中国现代中药材产业基地。让健康美味的商洛特产端上全国人的餐桌，给商洛百姓换回更多的收入！

（四）以生态建设和精品景区为抓手，打好全域旅游攻坚战。抓住西商一体化机遇，紧盯西安乃至关中上千万人口，充分利用我市绿水青山生态资源，大力发展避暑躲霾、休闲度假、民宿康养等新兴产业，积极实施景区品质提升、旅游要素完善、精准宣传营销、基础设施提质等行动，着力创建国家旅游休闲度假集中区和全域旅游示范市，把旅游业培育成我市新的战略性支柱产业。到2021年，全市4A级以上景区达到16个，较2016年翻一番，接待游客6000万人次、综合收入400亿元。让我们张开双臂，欢迎和拥抱更多的游客来我们商洛享受吧！

（五）以“一体两翼”和特色小镇为抓手，打好新型城镇化攻坚战。突出规划引领，强化产业支撑，做强“一体”、提升“两翼”，推动中心城区“东进、西延、南扩、北上、中改”，加快丹凤、洛南撤县设区，促进人口和产业协同聚集，使“一体两翼”成为最具活力的核心板块。统筹区域协调，推动山阳、商南撤县设市，促进镇安、柞水协同发展。集中打造20个左右的秦岭特色小镇，巩固提升重点镇、文化旅游名镇和美丽乡村建设成果，形成“一镇一特色、一村一景观、一路一风情”的新型城镇格局，到2021年，全市户籍人口城镇化率达到65%左右。使我们的城市有山有水有灵气、有花有草有生气、车水马龙有人气，让我们城市的环境更加舒适，农村的生活更加美好！

未来五年，我们还要着力抓好打基础、利长远、惠民生的十件大事：一是积极培育10家上市企业；二是确保西武高铁建成投运；三是推动商洛高新区创建为国家级高新技术产业开发区；四是建设集城市旅游、文化、休闲、地产、商务、度假为一体的金凤新城；五是新增2-3个5A级旅游景区；六是打造丹江百里生态长廊；七是全力推动丹宁高速开工建设；八是开发山阳漫川关、柞（水）宁（陕）国家旅游休闲度假区；九是实现主城区集中供热；十是实现“气化商洛”县区全覆盖。

打赢五大攻坚战，办成十件大事，将加快补齐我市发展短板，同步推进社会治理、民生保障等各个领域、各项事业快速发展，为建设创新美丽幸福商洛积蓄后发优势。

三、2017年重点工作

2017年是我市深入实施“十三五”规划的关键一年，也是新一届政府开局奠基之年。我们将牢牢把握“稳中求进”工作总基调，紧盯“追赶超越”目标，深入推进供给侧结构性改革，全面做好稳增长、促改革、调结构、惠民生、防风险各项工作，确保经济社会健康协调发展，以优异成绩迎接党的十九大胜利召开。

主要预期目标是：生产总值增长10%左右，地方财政收入增长6.5%，全社会固定资产投资增长16%，社会消费品零售总额增长12.5%，城乡居民收入分别增长9.5%和10%，居民消费价格指数控制在3%以内。

我们将立足当前，着眼长远，从事关商洛经济社会发展最基本、涉及人民群众利益最直接的事情着手，重点做好十项工作：

（一）坚持综合施策、精准脱贫，补齐全面小康“硬短板”

强力推进产业扶贫。持续推进现代农业产业精准扶贫示范市创建，全面推行“三带一创”产业精准脱贫模式，上半年编制完成贫困村综合发展规划，整合涉农资金，完善扶持政策，实施“一村一品”行动，扶持发展龙头企业、专业合作社，确保重点产业有“龙头”，村村都有合作社，产业脱贫户参与率70%以上。加强普惠型金融产业扶贫和开发性金融支持脱贫攻坚工作，大力推广“产业贷”“农户贷”“税信贷”等金融产品，扩大扶贫贷款担保基金规模，各县区不少于3000万元，全市新增扶贫贷款10亿元以上，抓好国开行支持脱贫攻坚一期35亿元项目实施。

全面决战移民搬迁。把今年作为移民搬迁“决战年”，落实移民搬迁新政策，坚持以集中安置为主，按照“四避开、四靠近、四达到”要求，以户定建、以产定搬、以人定扶、以户定业，全面完成“十三五”剩余5.5万户18.2万人的易地扶贫搬迁任务，同步完善基础设施和产业配套，确保搬迁对象搬得出、稳得住、能致富。

严格落实精准要求。以贫困户和贫困人口为基本单元，进一步夯实帮扶责任，找准脱贫路径，做到扶贫对象逐户核实、扶贫措施逐件落实、扶贫标准逐项检查、扶贫资金足额到位，确保每一户贫困家庭、每一名贫困群众，公平、均等地享受扶贫政策。建立健全第三方评估和贫困退出机制，强化监测督查，严格审核把关，同步构建返贫防范体系，完善政府救助、社会救助和保险救助“三位一体”救助机制，推进脱贫与低保有效衔接，强化社保兜底，防止因学、因病、因灾致贫返贫。全年实现16.97万贫困人口稳定脱贫、332个村脱贫摘帽。

（二）坚持项目带动、招商引资，培育跨越发展新动能

突出抓好重大项目建设。实施市级重大项目160个，总投资2019亿元，完成年度投资473亿元。强力推进70个续建项目建设，突出抓好镇安抽水蓄能电站、商洛电厂、山柞高速、必康医药产业园三期等投资超过50亿元的重大项目，确保30个以上项目建成投运。切实抓好90个新建项目建设，力促西武高铁、金凤新城、秦岭特色小镇等投资过百亿元的重大基础设施项目加快建设。扎实推进西渝高铁、榆商高速、商丹园区新医药产业综合开发等150个重大项目前期工作，力争50个项目具备建设条件。围绕城市建设、工业经济、全域旅游和社会民生等领域，策划论证一批大项目，市级重大项目储备不少于300个，各县区不少于100个。严格落实重大项目包抓责任制，实行项目资金动态管理，鼓励支持“大干快上”，推动重大项目落地。

切实加大招商引资力度。把招商引资作为推动发展的突破口和“助力器”，创新方式方法，在长三角、珠三角、京津冀等重点区域设立招商机构，承接东部产业转移，紧扣“334”十大优势产业集群，紧盯国际国内500强、行业领军企业和大型企业,做到招大招强招优、引资引智引技。大力改善投资环境，鼓励支持商洛籍在外创业成功人士返乡创业。夯实县区和部门的工作责任，强化考核评比机制，层层传导压力。重点抓好商洛万达广场城市综合体、商州药用植物提取智慧工厂、洛南维维集团核桃深加工、丹凤石墨资源开发、商南国漆科技产业园、山阳必康医药产业园、镇安钨钼产业园、柞水西川国际慢城、商丹园区新能源汽车全产业链等一批重大招商项目落地。全年招商引资到位资金525亿元，各县区至少有2个10亿元以上的招商项目落地。

创新推动投资融资工作。用好贫困地区IPO“绿色通道”政策，协助陕西盘龙药业尽快通过证监会审核上市，力争“新三板”挂牌企业实现“零”的突破，帮助5家以上优质企业在区域股权交易市场挂牌融资。完善政府和社会资本合作模式，抓好首批PPP示范项目建设，设立城市建设和产业发展基金，支持城投、交投等五家市级国有融资担保企业及各县区平台公司扩大资本金规模，多渠道破解发展资金难题。

（三）坚持产业兴市、转型发展，打造经济发展新引擎

力促工业稳定增长。落实省市工业稳增长促投资政策措施，强化各类要素保障，加强运行监测分析，确保陕西锌业、尧柏水泥等骨干企业满负荷生产。支持跃迪电动客车、必康医药产业园二期、比亚迪电池材料等20个重大建成项目产能释放。充分利用在线创客平台，创新企业销售模式，工业产销率达到90%以上，新增规模以上企业25户，规模以上工业增加值增长14%。

推动工业转型升级。加速传统工业和信息化深度融合，全面落实“中国制造2025”商洛实施方案，大力发展新材料、新能源、电子信息等战略性新兴产业。加大技改投入和新产品研发，推进绿色制造，扩大先进产能，促进矿产建材、食品加工等传统产业转型升级。实施30个重点技术改造项目，抓好秦煌10万辆电动汽车、香菊鼻科新产品研发、商州森弗大健康产品、商南3C数码电子产品等50个市级重点工业项目建设，完成投资85亿元。

提升园区承载能力。启动商洛高新区创建国家级高新技术产业开发区工作，加快山阳工业集中区创建省级高新区，持续抓好15个县域工业集中区扩容提质，重点建设科技研发、信息物流、电子商务、质量检测等服务平台，着力完善供水、供电、供气、排污等基础设施，清理闲置用地，处置僵尸企业，拓展发展空间。理顺园区管理体制，增强服务功能，扩大省级小微企业创业创新示范基地建设规模，新建标准化厂房20万平方米，新增入园企业40户，着力在融资模式创新、重大项目建设、先进技术引进、优质品牌打造上实现新突破。

发展民营经济和现代服务业。认真落实中省市支持民营经济加快发展的若干政策措施，放宽投资限制，支持民间资本参与道路交通、水电燃气、供热供暖等基础设施PPP项目建设。实施中小企业培育成长计划，扶持龙头企业、“瞪羚企业”、高新企业发展，培育一批规模以上民营骨干企业。大力发展民宿休闲、体育健身、养老家政、医养结合等现代服务业，新增限上商贸企业15家，服务业增加值增长10%。全年新增各类市场主体7500户、个体工商户5000户、私营企业500户，非公经济增加值占GDP比重达到56%以上。

不断壮大县域经济。统筹推进撤县设区、设市工作。抢抓建设国家生态文明综合改革示范区的机遇，走绿色循环发展路子，促进产业集约化、市场化发展。支持县域经济发展，调整优化经济结构，培育优势产业，壮大骨干财源。鼓励县区争先进位，提升县域经济综合竞争力。

（四）坚持品牌引领、三产融合，提升发展特色现代农业

推进农业供给侧结构性改革。以建设秦岭生态农业示范市为抓手，提升优质农产品生产能力，加大绿色优质农产品供给。新发展核桃示范园7万亩，板栗园2万亩，茶园3万亩，中药材基地30万亩，代料食用菌1.7亿袋，种植马铃薯90万亩，粮食总产稳定在50万吨左右。创新农业经营机制，促进农业适度规模经营。推行绿色标准化生产，确保化肥、农药零增长，争创国家农业可持续发展试验示范市。

做实叫响商洛山地农产品牌。大力开展品牌创建行动，认证“三品一标”25个，鼓励企业争创知名商标20个以上，积极创建“商洛核桃”“商洛香菇”“镇安板栗”“秦岭泉茗”等一批有影响力的区域公用品牌。加大现有品牌应用管理，用好“国家农产品质量安全市”金字招牌，健全农产品质量安全追溯体系。全方位推介宣传农产品优势品牌，让“商洛山地农产”品牌唱响全国、走向世界。

加快现代农业园区建设。做精县级园区、做大市级园区、做强省级园区，全年认定省、市级现代农业园区10个以上。实施全流域开发，建设精品园区集群，加快推进农产品加工园区建设，重点打造柞水西川、丹凤棣花、商南试马等8个特色现代农业园区，使园区成为现代农业发展的新引擎。

推动一二三产融合发展。积极培育新型农业经营主体，重点发展加工型、营销型龙头企业，力争每县区有1-2个地方领军型龙头企业，认定市级以上龙头企业10个以上，农产品加工转化率达到40%以上，支持各县区争创全省农业产业转型升级示范县。加快打造区域性农产品贸易集散中心，支持洛南建设西北核桃交易市场、商南建设食用菌交易市场、镇安建设板栗交易市场。创建国家农村电子商务示范市，发展农村电商服务站点100个，电商交易额增长20%以上，力促特色农产品“上网、入市、销全国”。大力发展观光农业、休闲农业、创意农业，开辟农村发展新路径、拓展农业增效新空间、拓宽农民增收新渠道。

（五）坚持以人为本、城乡统筹，加快推进新型城镇化

完善城市规划体系。织密基础专项规划网底，填补公共专项规划空白，编制完成中心城区主要街路绿地系统改造、集贸市场、公共停车场、地下综合管廊等专项规划，建成城市规划展览馆。加快各县城总体规划修编和专项规划、控制性详细规划编制工作，详规覆盖率达到90%。完成“五规合一”重点镇规划编制。严格城市规划执法，使规划成为不能逾越、不敢逾越的“红线”。

推进城市扩容提质。投资195亿元，实施中心城区“四大门户区”和国家级丹江水利风景名胜区改造提升，启动312国道构峪至麻街岭城市道路改造，加快推进环城南路、丹南新区路网、南秦新区、北新街东延、候塬棚改和丹凤环城北路建设等95个项目，“一体两翼”城市建成区面积达到60平方公里、人口达到58万人。以棚户区改造为抓手，加强四县县城公共设施和服务配套，建设一批协调城市景观风貌、体现“商洛符号”的标志性建筑。支持山阳、丹凤开展全国、全省新型城镇化综合试点。积极推进有条件农民进城落户，全市户籍人口城镇化率达到53.5%。

改善城市人居环境。以“四城联创”为抓手，实施中心城区绿化工程，中心城市绿化覆盖率达到36％、绿地率达到31％、人均公园绿地面积8平方米。实施北新街、江滨大道、龟山北坡和机关单位、大型商业综合体、住宅楼楼体的亮化工程，打造多彩“不夜城”。启动中心城区集中供热工程，建成海绵城市示范点6处，地下综合管廊16公里。加快“智慧城市”建设，市县区全部建成数字化城市管理平台。加快城市综合执法体制改革，推进执法监管重心下移。

创建秦岭特色小镇。按照“特色产业+文化+旅游”的发展模式，重点抓好7个以上特色小镇建设。持续推进沙河子、竹林关2个省级重点示范镇，漫川关、凤凰、云盖寺、棣花等4个省级文化旅游名镇，巡检、营盘、高坝、杨峪河等17个市级重点镇建设。推进产景、文旅和多业融合，打造商洛乡村旅游升级版，让山里人过上比城里人更美好的生活。

加强城乡基础设施建设。投资145亿元，实施国省干线公路改造提升、农村公路建养、农村水电讯等45个城乡基础设施建设项目，做好丹宁高速前期准备工作，创新推动商州、镇安、柞水普通公路“建养一体化”试点。加快“气化商洛”商州至洛南、商南天然气长输管线建设，力争商州至山阳段开工，做好西安至镇柞段前期工作。实施农村电网改造升级，抓好云镇水库、磨沟水库、清油河水库等重点水利水源项目建设，推动城镇供水管网、通信基础设施向农村延伸。

（六）坚持生态立市、绿色发展，保护好商洛赖以生存和发展的绿水青山

加大环境治理力度。启动实施丹江、洛河、金钱河、乾佑河等流域污染防治行动计划，加强水源地保护，抓好市第二污水处理厂前期、商丹园区工业污水处理厂建设和6县区污水处理厂提标改造，中心城市垃圾、污水无害化处理率分别达到96%、88%，县城分别达到91%、83%。加强农村环境综合整治，强化面源污染治理，确保出境断面水质稳定达标。全面淘汰黄标车，建立市环境监控应急指挥平台，市区空气质量优良天数保持在300天以上。实行能源、水资源、建设用地总量和强度双控制度，推广高效节能和清洁生产技术，全面完成节能减碳减排任务。

提高生态建养水平。围绕创建全国循环经济示范市，抓好4大类32个循环经济重点项目建设，确保10大类74项评价指标全部达标。抓好“两岸三线四区”重点区域造林绿化，深入实施天然林保护和退耕还林等工程，完成植树造林25万亩。实施丹江干流防洪工程，启动丹江百里生态长廊建设，治理水土流失700平方公里。强化国土资源管理，做好矿产资源保护和综合开发，推进绿色矿山建设。加大基本农田保护力度，加强土壤污染防治。

整治环境突出问题。全力配合中省环保督察巡查，扎实开展全市环保督查巡查，狠抓环境突出问题的查处整改，严厉打击乱砍滥伐、乱采滥挖、非法占地等违法行为。严格落实政府属地管理责任、部门监管责任、企业主体责任，开展领导干部自然资源资产管理和环境保护责任离任审计，建立环境信访案件常态化办理机制，做到“不查清不放过、不处理不放过、不整改不放过”。

（七）坚持依托生态、发展全域旅游，把绿水青山变成强市富民的金山银山

全面启动全域旅游创建。编制《商洛市全域旅游发展规划》，出台《创建全域旅游示范市实施意见》，以创建全域旅游示范市带动各县区创建全域旅游示范区，推动景区景点建设提升。压实责任，强化考核，力争商南、柞水通过国家全域旅游示范区验收命名，山阳通过省级初验，镇安纳入创建序列。

抓好现有景区提档升级。启动漫川关国家旅游休闲度假区建设，做好柞宁国家旅游休闲度假区规划编制和前期工作，推进牛背梁、天竺山5A和金台山、九天山等4A级景区创建，巩固提升美丽乡村建设成果，加快蟒岭绿道、柞水终南山寨、洛南仓颉文化艺术园、商於古道二期、商南金丝峡旅游产业园、塔云山景区三期开发等项目建设，各县区年内新增3A级景区各1家，全市新增4A级景区2家。加快市县区旅游咨询服务中心、智慧旅游服务平台建设，提升旅游服务水平和游客满意度。

实施“旅游+”发展战略。推动旅游+城镇化、旅游+文化、旅游+工业、旅游+农业、旅游+康养等产业融合发展，加大旅游商品、特色餐饮研发推广和民俗风情、文化演艺挖掘展示，培育高端民宿，发展养生养老和康体度假。瞄准西安及周边地区一级客源市场，精心策划举办2017秦岭生态旅游节、千万人游商洛、秦岭与黄河对话及游最美奇峡、揽牛背花海、享漫川生活、赏千亩荷塘等十大品牌旅游活动，加大宣传营销力度，把大家的眼球和注意力吸引到商洛来，实现月月有亮点、季季有高潮，年接待游客达到4100万人次、旅游综合收入225亿元以上。

（八）坚持创新驱动、深化改革，为追赶超越增添新活力

推进供给侧结构性改革。认真落实“三去一降一补”政策，完成省上下达的去产能任务。加大开采、选冶等小矿点、粗加工、短链条、低附加矿产企业整合改造提升，优化钼、钒等优质矿源、重点领域的产能结构。加强房地产市场监管，提高棚改货币化安置率，中心城市不低于80%、各县不低于60%，全年完成商品房去库存6500套。落实国家减税政策，制定出台企业降费措施，清理规范各类涉企收费，切实降低企业成本。

深化农村综合改革。全面实施农村土地所有权、承包权、经营权“三权分置”改革，统筹推进农村土地征收、集体经营性建设用地入市、宅基地改革、农民住房财产权抵押贷款试点，赋予农民更加充分的财产权利。积极推进农村集体产权制度改革，认真搞好集体资产清产核资，有序推进经营性资产股份合作制改革，搭建农村集体产权流转交易平台，发展壮大集体经济。开展土地承包权有偿退出、经营权抵押贷款试点，支持新型农业经营主体、社会化服务组织开展土地经营权流转、股份经营、托管经营等多种形式的规模经营。

推进重点领域改革。持续推进“放管服”改革,再取消、下放、调整一批“含金量”高的审批事项，深化“多证合一、一照一码”和“证照分离”改革，全面推行“双随机一公开”，进一步加强以信用为核心的市场监管体系建设。推进投资项目在线联网审批，加快市级公共资源交易平台建设。持续深化国企国资改革，完善现代企业制度。实施产权制度改革，加强对各种所有制组织和自然人财产权的保护。抓好财税、金融、土地、供销、标准化等基础性重大改革。

推动创新开放融合发展。积极促进大众创业万众创新，抓好创新型商洛建设，加大科技研发投入，支持中国地质大学知识产权与技术转移商洛分中心发展，加快柞水创建全国科技创新驱动县域经济发展示范县，全市建立企业工程技术研发中心15个。完善创业服务体系，全年打造众创平台10个以上，发放创业扶持贷款2亿元。加快陆路货运口岸建设，创建省级特色出口示范基地2个，外贸进出口总额增长10%。

（九）坚持公平共享、和谐稳定，全面发展城乡社会事业

健全完善社会保障体系。做好以高校毕业生、退役军人、城镇就业困难人员、去产能分流职工为重点的就业工作，开展劳动技能免费培训2.1万人次，新增城镇就业1.5万人，转移农村劳动力就业40万人。推动养老保险制度改革，稳步提高社保待遇，继续推进城乡医保整合，启动实施异地就医住院医疗费用直接结算。加强特困人员救助工作和农村特困人员供养机构建设，搞好政府购买社会救助试点工作，实现社会救助规范化镇村创建全覆盖。加快市中心老年公寓和各县老年公寓建设，鼓励社会资本参与城乡养老事业。新建社区日间照料中心8个、农村互助幸福院150个，深入推进殡葬改革。加强农村留守人员关爱保护，落实残疾人补贴政策，抓好市残疾人康复中心建设。

提高健康服务水平。搞好综合医改和城市公立医院改革试点，推动优质医疗资源下沉，加快5个县级医院改扩建，推进镇（办）卫生院及社区、村级卫生室标准化建设，加大卫生技术人才引进力度，患者县域内就诊率达到90%以上。建成市级计划免疫和新农合信息系统。巩固药品“三统一”成果，县级以上医疗机构全部实行药品质量追溯制度。加快中医药产业发展，建设60个镇级中医馆，创建3个全国基层中医药先进单位。全面落实计生奖励优惠政策，实施孕产妇系统保健免费服务项目，人口自增率控制在6.3‰以内。

发展教育文化体育事业。推进教育均衡发展，加快义务教育学校标准化建设，不断改善办学条件，实施教育建设项目22个，全年完成投资10.48亿元。认真落实贫困县乡村教师生活补助政策，推进初高中未升学毕业生技能培训全覆盖。启动实施第三期学前教育行动计划，扶持普惠性民办幼儿园发展。深化学区制管理改革，严格实行义务教育免试就近入学政策。推动洛南“双高双普”创建达标。支持特殊教育、高等教育、民办教育健康发展。完善公共文化服务体系，加大艺术精品创作力度，传承红色基因，讲好“商洛故事”，推进镇办综合文化中心、农村新型社区农家书屋、文化活动室建设，启动建设商洛市博物馆，做好第八届省艺术节和第三届现代文化艺术节参演工作，提高文化软实力。启动全国“十四运”女子排球、公路自行车等赛事场馆改建，高标准办好2017环秦岭公路自行车赛、陕西·商洛首届马拉松赛、四国男篮挑战赛等十大精品赛事。

全面提升社会治理水平。加快建设城乡社区服务中心和社区公共服务综合信息平台，推进社区、社会组织、社会工作者和社会志愿者“四社联动”。建立“党政同责、一岗双责、失职追责”的安全生产责任体系，严格落实各级党委、政府领导责任和部门监管责任、企业主体责任，坚决遏制重特大事故发生。积极落实“四个最严”要求，加强食品药品监管能力建设，建成市级食品检测检验中心，推行食用农产品合格证制度。抓好平安商洛建设，加强社会治安综合治理，加大对非法集资、传销等违法犯罪活动的打击力度。全面推进“七五普法”，建立矛盾纠纷多元化解和风险防范机制，持续推进法治信访、责任信访、阳光信访，全面开展信访“三无”县区创建。完善社会信用体系，合力共建“信用商洛”。坚决拥护支持军队改革，扎实做好国防动员、兵役征集、国防教育、军事设施保护、人民防空、民兵预备役、军民融合和双拥共建等工作，切实抓好军转干部安置、复退军人就业和贫困军属优抚政策的有效落实。

（十）坚持服务群众、民生为先，努力办好十件实事

一是缓解中心城区“上学难”问题。力促新建商中、市初级中学、商州区思源学校和嘉园国际、商丹高新幼儿园建成投用，加快推进秦韵教育城、商州区高级中学，商州区第六、第十五小学，商州区第四、第十二、第十三幼儿园建设，改扩建商州区第一、第三小学。全年新增高中学位1000个、初中学位4800个、小学学位1000个、幼儿园学位1200个。

二是解决企业和群众“办事难”问题。建成市级政务服务中心，同步推进县区政务服务中心标准化建设，整合部门信息，建立资源共享机制，实现市县区联网运行，同步建成网上大厅，对所有行政审批和服务事项实行“一厅式办理”。

三是缓解中心城区“停车难”问题。充分利用现有闲置的地下停车场，建成东关老体育场、丹江公园等6个地上、地下停车场和立体停车场，新增泊车位5000个。

四是实施中心城区公共交通改善工程。积极推进出租车行业改革，理顺城市公交经营体制，更新增加城市公交数量，扩大公交线路覆盖面，推行刷卡乘车，加快城东客运站建设。

五是实施中心城区市场建设工程。新建4个、改造提升2个市场，建成2个小吃城，下茬整治北新街、中心街、东环路、团结路、东关市场的乱摆乱放及占道经营问题。

六是实施中心城区清洁保洁提升工程。新建改造下沉式垃圾转运站17座、垃圾收集屋58座，购置各类垃圾清运、清扫车辆70辆，推行垃圾分类回收，减少二次污染。实施背街小巷改造提升，建设二类公厕61座，改造城中村、城乡结合部旱厕3000座。

七是实施中心城区健身休闲场所完善工程。开放市体育馆，建设商洛游泳馆和丹江、江滨银杏公园健身示范长廊，建设6个小广场，改造提升金凤山、龟山公园。

八是实施中心城区城周绿化工程。按照改善城市生态、做美城市、增绿添彩的要求，全面抓好市区直观坡面造林绿化，完成高标准造林2万亩，确保三年实现裸露山体全部绿化达标。

九是实施中心城区文化惠民工程。建成市图书馆、群艺馆、工人文化宫、青少年活动中心、妇女儿童活动中心。组织中心城区群众性惠民演出20场次。

十是实施中心城区河道综合治理工程。完成黄沙河、柳家沟、马莲峪河道综合治理，彻底解决污水、垃圾等环境污染问题。

各县区政府也要围绕上学、就医、就业、健康、养老、出行等民生民本问题，办好事关人民群众切身利益的实事、好事，市政府将从各方面给予大力支持。

四、全面加强政府自身建设

各位代表，实施追赶超越，历史重任在肩，全面建成小康，使命神圣光荣。加强政府自身建设，是全面从严治党的要求，是时代的召唤，更是人民群众的热切期盼。我们将围绕树立“五个理念”、强化“五个意识”，切实加强作风建设，确保今年各项任务圆满完成和今后五年宏伟目标如期实现。

（一）牢固树立执政为民理念，不断强化服务意识。各级政府都必须时刻牢记，我们的政府是人民的政府，我们的权力是人民赋予的，为人民服务是政府的天职。人民对美好生活的向往，就是我们的奋斗目标。政府全体工作人员都是人民的勤务员、服务员、办事员，要下决心根除群众办事“门难进、脸难看、事难办”现象。我们绝不容许政府公务人员“当官做老爷”！

（二）牢固树立依法行政理念，不断强化敬畏意识。依法行政是法治政府的本职要求。各级政府工作人员都必须始终怀着如履薄冰的心态，时刻心存敬畏，敬畏法律、敬畏权力、敬畏人民，做到严以用权、慎于用权。坚决摒弃“政府万能”的观念，政府的一切行为都必须限制在法治的框架之内，都必须置于党的领导和人大、政协、群众的监督之下。政府一切权力都必须关在法律和制度的笼子里，在阳光下运行。我们绝不允许以权代法、以权压法的现象发生！

（三）牢固树立改革创新理念，不断强化担当意识。实现市第四次党代会提出的建设创新美丽幸福商洛的宏伟目标，打赢精准脱贫攻坚战、全面建成小康社会，任重而道远。我们必须锐意改革、大胆创新，以“跳起来摘桃子”的进取精神积极作为。每一位政府工作人员，都必须强化担当意识，敢啃硬骨头、敢为人之先，坚决杜绝懒政、庸政、怠政行为，切实提高行政效能。我们绝不给不作为、慢作为、尸位素餐的人留位子、留空间！

（四）牢固树立为政清廉理念，不断强化廉洁意识。习近平总书记常讲：“打铁还需自身硬”。古人说，公生明、廉生威。作为政府工作人员，要赢得人民群众的信任，首先必须管住自己的手、管住自己的嘴、管住自己的腿、管住自己的人，严守“八项规定”，保持两袖清风。当官就不要想发财，发财就不要想当官。我们绝不给既想当官、又想发财的人提供任何机会！

（五）牢固树立苦干实干理念，不断强化落实意识。“一分部署、九分落实”，为政之道、贵在落实。我们要以落实市委决策和人大决议为己任，以苦干实干的精神抓落实，以务实求细的态度促落实，以严格考核严肃问责的措施保落实，撸起袖子加油干，精文减会、多抓落实，坚持不懈、久久为功，把宏伟蓝图变为美好现实，把奋斗目标变为人民群众的幸福指标，向全市人民交出一份满意的答卷！

各位代表，人民重托使命如山，追赶超越时不我待。让我们紧密团结在以习近平同志为核心的党中央周围，在省委、省政府和市委坚强领导下，汇聚正能量、提振精气神，锐意进取，攻坚克难，为建设创新美丽幸福商洛而努力奋斗！
